# *Poa secunda* local collections and commercial releases: A genotypic evaluation

**DOI:** 10.1371/journal.pone.0173221

**Published:** 2017-04-03

**Authors:** Alanna N. Shaw, Daniel L. Mummey

**Affiliations:** MPG Ranch, Missoula, Montana, United States of America; National Cheng Kung University, TAIWAN

## Abstract

The genetics of native plants influence the success of ecological restoration, yet genetic variability of local seed collections and commercial seed releases remains unclear for most taxa. *Poa secunda*, a common native grass species in Intermountain West grasslands and a frequent component of restoration seed mixes, is one such species. Here, we evaluate the genetic variation of local *Poa secunda* collections in the context of wild populations and commercial seed releases. We evaluated AFLP markers for seven *Poa secunda* collections made over a 4000-hectare area and four commercial releases (High Plains, MT-1, Opportunity, and Sherman). We compare the genetic distance and distribution of genetic variation within and between local collections and commercial releases. The extent and patterns of genetic variation in our local collections indicate subtle site differences with most variation occurring within rather than between collections. Identical genetic matches were usually, but not always, found within 5 m^2^ collection sites. Our results suggest that the genetic variation in two *Poa secunda* releases (High Plains and MT-1) is similar to our local collections. Our results affirm that guidelines for *Poa secunda* seed collection should follow recommendations for selfing species, by collecting from many sites over large individual sites.

## Introduction

*Poa secunda* is a cool-season perennial bunchgrass common to grasslands of western North America. A polyploid complex, *P*. *secunda* is composed of variants that may have distinct ecological and biogeographical roles [1], though they are polymorphic variants of a single species [2,3] with a ploidy range from 2n = 42–100 [4–7]. These variants include big bluegrass (*‘*Ampla*’*), Canby’s bluegrass (‘Canbyi*’*), Pacific/slender bluegrass (‘Gracillima’), Nevada bluegrass (‘Nevadensis’), alkali bluegrass (‘Junctifolia’), Sandberg bluegrass (‘Sandbergii’), and pine bluegrass (*‘*Scabrella’) [1,8]. *Poa secunda* is a facultative apomict, reproducing via selfing, outcrossing, and apomixis [3,9,10]. Apomixis is pseudogamous in *P*. *secunda* [10], so pollination is biochemically necessary for seed development, but is not associated with paternal inheritance [11]. *Poa secunda* individuals often produce viable pollen and can contribute to selfing, outcrossing, and apomictic seed development even if they are entirely or highly apomictic [10].

Despite the common belief among growers and restoration practitioners that selfing and outcrossing are rare in *P*. *secunda* and that the species is predominantly apomictic [12], the relative prevalence of reproductive modes in *P*. *secunda* is unknown. Kellogg [10] demonstrated that apomixis varies in frequency in *P*. *secunda* at the individual (up to 40% apomictic ovules) and population (25–100% apomictic ovules) levels, as well as with site conditions. Kelley et al. [7], however, found apomictic seed development in six out of seven *P*. *secunda* wild-collected seed lots, suggesting high levels of apomictically generated seed. Clausen et al. [13] and Kellogg [10] established that *P*. *secunda* does outcross at a fairly low rate, averaging 0.46% and 0.6%, respectively, though the former characterized hybrids as poor performers.

Patterns of gene flow influence genetic variation across the landscape. Genes translocate and/or recombine by outcrossing, selfing, and asexual reproduction [14–16] such that landscape genetic structure depends on the distance of pollen exchange and/or seed dispersal, as well as environmental constraints to seedling establishment and persistence. In asexual plants, dispersal and environmental selection structure plant genotypes, leading to a patchy mosaic of genetically similar plants [11,17–20]. When plants self-pollinate, populations diverge over time as they lose heterozygosity and experience environmental selection [14–16,21]. Outcrossing plants form less spatially structured and more heterogeneous populations [14–16].

Patterns of genetic variation are difficult to predict in plants with mixed reproductive modes, such as *P*. *secunda* [22,23]. Even infrequent outcrossing in species that primarily self-fertilize can cause patterns of variation resembling outcrossing species [14,24,25,26], while apomictic reproduction increases the risk of under-sampling genetic diversity. Recommendations for germplasm collection are often based on species characteristics such as life history and mating system [27]; to capture genetic diversity efficiently, collection programs benefit from knowledge of population genetic structure.

Gene flow depends on propagule dispersal that, in grasses, is mostly localized with rare long distance dispersal events [28,29]. Propagules must then germinate, establish, and persist to reproductive maturity; the environment thus constrains landscape genetics in plants. Introduced germplasm in restoration plantings often results in translocation over distances much greater than expected in wild populations. This practice risks negative impacts on population fitness, including inbreeding and outbreeding depression resulting from the introduction of limited diversity or divergent/maladapted genotypes, respectively [30]. At the same time, these risks must be weighed in light of plant communities that have undergone novel anthropogenic alterations and/or invasion by exotic species [31].

Commercial seed releases are often used in restoration plantings [32–35]. Local plant populations and commercial plant materials experience different selective pressures [36,37]. Directional selection reduces the effective population size of releases [36] and seed increase processes provide myriad opportunities for genetic truncation via selection and drift [38–41]. Although *P*. *secunda* has a capacity for apomictically “fixed” genotypes, as in the Sherman (single genotype) and Canbar (three genotypes) releases [42], the genetic consequences of seed increase in *P*. *secunda* are unknown.

We evaluated local *P*. *secunda* collections to assess small-scale genetic variation and to inform germplasm sampling strategies. We also examined four *P*. *secunda* releases to compare their genetic variability and evaluate their suitability for use as plant materials in nearby restoration sites. Our study is the first to compare genetic variation and structure in *P*. *secunda* local collections and commercial releases. We examined this common bunchgrass species, which shares an early spring phenological niche with the important invasive species *Bromus tectorum* with the aim of broadening native plant genetic information to inform restoration management. In particular, we predicted that: 1) local collections are moderately variable given previous studies of phenotypic variability [43,44]; 2) commercial releases differ in their genetic identities and level of variation as a function of variant identity, geographic distance between source collection sites, and sourcing paradigm; and 3) local collections will differ significantly from commercial releases as a function of geographic distance between source collections and local site and variant identity.

## Methods

### Plant materials

**Fig 1:**
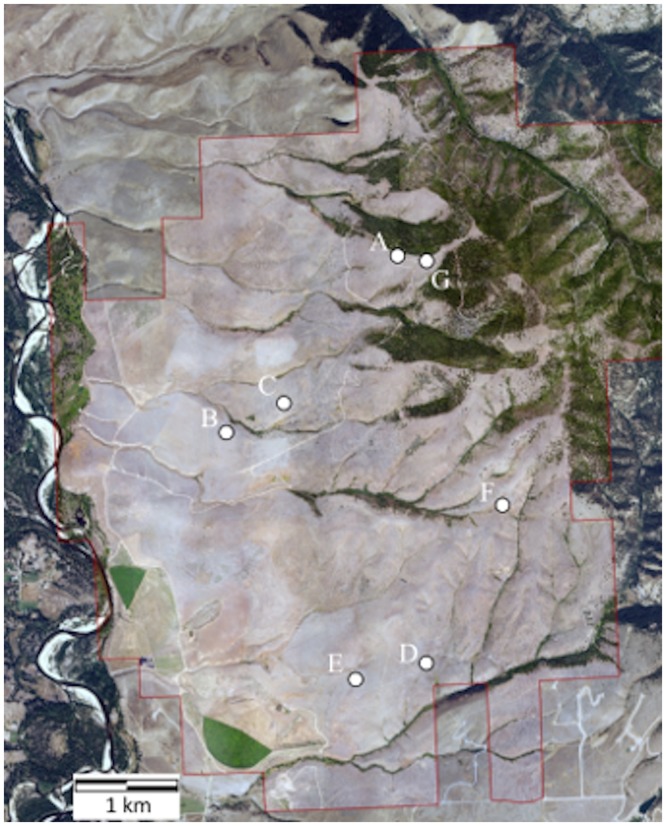
Local *Poa secunda* collection sites. *Poa secunda* collection sites are indicated by white dots and letters. Approximately 20 plants were collected from each site. The red line outlines the boundary of MPG Ranch.

*Poa secunda* individuals (‘Sandbergii’) were collected from seven sites located on MPG ranch, a private 4,000-hectare conservation property near Florence, MT (11T, 728907.423, 5175718.659; Fig 1) with permission from the landowner. Green tissue was clipped from approximately 20 randomly selected local plants at each 5 m^2^ collection site. Additionally, we evaluated four commercial releases of *P*. *secunda*, including High Plains, MT-1, Opportunity, and Sherman (Table 1). The High Plains germplasm was developed from three collections made in Natrona, Co. (1980), Campbell Co. (1980), and Uinta Co. (1983), WY, and released as a selected class germplasm in 2000 [45]. The MT-1 germplasm was developed from collections made in Toole, Co., MT in 1996–1997 and released as a source-identified plant material in 2008 (Western Reclamation, Eltopia, WA, USA). Both High Plains and MT-1 represent the ‘Sandbergii’ variant. Opportunity (‘Nevadensis’) germplasm was developed from a collection made in a mine-contaminated site east of Anaconda, MT in 1998 and released as selected glass germplasm in 2007 [46]. Sherman (‘Ampla’) was increased from a collection made in Moro Co., OR in 1932 and released in 1945 as a cultivar [47]. Commercial *P*. *secunda* releases (Table 1) were grown from seed in 66 mL Ray-Leach cone-tainers (Stuewe & Sons, Tangent, OR, USA) under grow lights to obtain tissue for genetic analysis. All local and commercial tissue samples were frozen (-20°C), lyophilized, and pulverized by bead beating prior to DNA extraction.

**Table 1:** *Poa secunda* commercial release source information.

Release	Variant	Geographic origin (collection date)	Plant material type (release date)
**MT-1**	‘Sandbergii’	Toole Co., MT (1996–1997)	Source-identified seed (2008)
**High Plains**	‘Sandbergii’	Natrona Co., WY (1980) Campbell Co., WY (1983) Uinta Co., WY (1980)	Selected class release (2000)
**Opportunity**	‘Nevadensis’	East of Anaconda, MT (1998)	Selected class release (2007)
**Sherman**	‘Ampla’	Moro, Sherman Co. OR (1932)	Cultivar (1945)

### Molecular methods

We extracted total DNA from ~10 mg of pulverized, lyophilized leaf tissue using a CTAB-chloroform protocol modified for use in a 96-well format. The extracted DNA was quantified using a Quibet 2.0 fluorometer (Thermo Fisher Scientific Inc., Waltham, MA USA). We performed AFLP analysis according to Vos et al. [48] as modified by Blignaut et al. [49]. Modifications to Blignaut et al. [49] included: substitution of the restriction enzyme *Mse*I and corresponding adapters/primers for *Tru*I, use of GoTaq^®^ Colorless MasterMix (Promega, Madison, WI, USA), omission of DNA purification, and the addition of a second unlabeled, selective MseI+NNN primer (see Table 2).

**Table 2:** Adapters and primers used in AFLP genotyping.

Primer Name	Label	Sequence (5’-3’)
EcoRI-forward adapter	NONE	CTC GTA GAC TGC GTA CC
EcoRI-reverse adapter	NONE	AAT TGG TAC GCA GTC TAC
MseI-forward adapter	NONE	GAC GAT GAG TCC TGA G
MseI-reverse adapter	NONE	CTA CTC AGG ACT CAT
EcoRI+0	NONE	GAC TGC GTA CCA ATT C
MseI+0	NONE	GAT GAG TCC TGA GTA A
EcoRI+ATG	6-HEX (IDT)	GAC TGC GTA CCA ATT CAT G
EcoRI+CAT	FAM (IDT)	GAC TGC GTA CCA ATT CCA T
EcoRI+AAT	NED (AB)	GAC TGC GTA CCA ATT CAA T
MseI+CTT	NONE	GAT GAG TCC TGA GTA ACT T
MseI+CTC	NONE	GAT GAG TCC TGA GTA ACT C

Briefly, approximately 200 ng of DNA was digested with the rare cutter restriction enzyme *Eco*RI-HF (New England Biolabs, Ipswich, MA) and the frequent cutter *Mse*I (New England Biolabs), ligated to corresponding adapters with T4 DNA ligase (New England Biolabs), and diluted (1:10 v:v) with molecular grade dH_2_0. The restriction-ligation mix was PCR-amplified using *Eco*RI and *Mse*I pre-selective primers (Table 2; Integrated DNA Technologies (IDT), Coralville, Iowa, USA) without selective bases and diluted (1:19 v:v) prior to selective amplification. Selective amplification with six primer combinations followed, with EcoRI+NNN primers 5’ fluorescently end-labeled with FAM (IDT), 6-HEX (IDT) or NED (Applied Biosystems) and unlabeled MseI+NNN (see Table 2). Primer combinations were multiplexed with each unlabeled MseI+NNN primer separately. Polymerase chain reactions followed Blignaut et al. [49]. Approximately 15% of samples were replicated to evaluate reproducibility [50]. Additionally, the release Sherman, determined by Larson et al. [42] to be genetically uniform, was used as a positive control and molecular grade deionized water as a negative control.

Capillary electrophoresis was performed with 3730 XL DNA analyzers (Applied Biosystems, California, USA) using LIZ-500 (Applied Biosystems) and ROX-500 (Applied Biosystems) size standards for primer combinations 1–3 and 4–6, respectively (Table 2). Fragments between 50–300 bp were scored visually for presence or absence using GeneMarker V2 4.0 software (Softgenetics, State College, PA USA). Replicated samples were scored independently and profiles were compared to calculate reproducibility by dividing the number of scoring mismatches by the total number of AFLP markers.

### Data analyses

We calculated polymorphism within local and commercial sources by dividing the number of polymorphic bins (markers) that occurred at a rate greater than the error threshold (5%) by the total number of reproducible bins [50]. Monomorphic bins (including markers that were polymorphic at a level below the error threshold) were excluded from further analyses.

We constructed Jaccard distance matrices of polymorphic AFLP profiles and performed pairwise comparisons of average Jaccard distance of releases, within collections, between collections and overall for local collections. Samples with complete pairwise similarity with respect to the 5% error threshold were identified in the dataset using pairwise Jaccard similarity indices. To calculate the total percentage of identical genotypes, we divided the number of individuals with one or more identical genotype by the total number of individuals within each collection or release. Genetic identical match rates were calculated within and among local and commercial collections. Additionally, we calculated the overall number of identical genetic matches in combined local collections and the frequency of within and between collection genetic matches.

We performed overall comparisons of releases and collections using principle coordinates analysis with a Jaccard similarity index. We evaluated the relationship between local genetic and geographic variation and elevation using Mantel tests [51]. We used a digital elevation model, derived from Shuttle Radar Topography Mission data, from the USGS Earth Resources Observation and Science (EROS) Center (http://eros.usgs.gov) to estimate elevation on each site. To evaluate the importance of spatial distance, we constructed a Euclidean distance matrix from spatial coordinates of collection sites. Mantel tests were performed using PASSAGE software.

Analysis of molecular variance (AMOVA) [52,53] evaluated the proportion of variance within and among local collections and/or releases and was calculated using GenAlEx 6.5 [54,55]. Distance-based redundancy analysis (DB-RDA) [56] was used to evaluate variation among AFLP profiles in terms of collection and release identity. We used Bray-Curtis distance matrices and constrained each analysis by *P*. *secunda* source identity (9999 iterations) using Canoco (ver. 5) software.

## Results

### Polymorphism and error reporting

We scored 236 AFLP markers with an average error rate of 2.36%. Twenty percent of these markers were polymorphic among samples at a rate greater than the error threshold of 5%, resulting in 47 polymorphic markers, 19.9% overall polymorphism, and an error rate of 4.92%. Polymorphism rates varied between sources (local population or commercial release), with the proportion of polymorphic markers ranging from 0% (Sherman) to 30.93% (High Plains).

### Local variation of *Poa secunda*

**Fig 2:**
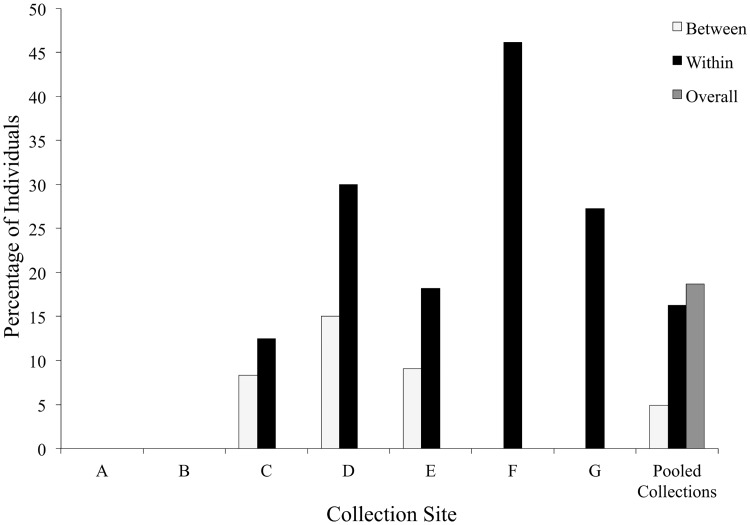
*Poa secunda* genetic matches within and between local collections. Percentage of *Poa secunda* individuals with one or more genetic match within (black bars) or between (white bars) local *Poa secunda* collections. Pooled collections indicate variation in genetic matches within and between all collections and the overall percentage of individuals with one or more genetic match (gray bar).

Distance-based redundancy analysis explained 14.3% of AFLP variation (*F* = 4.0; *P* < 0.002) among local collections (Table 3). Five out of seven collections had at least one individual with one or more within-collection identical genetic match (Fig 2). To evaluate the importance of identical genetic matches for between-site variation, we excluded samples with one or more within-collection genetic match and found that collection site explained 12% of variance (*F* = 2.2; *P* < 0.002), indicating that identical genetic matches explained only 2.3% of the variation among collection sites. Overall, 18.7% of local individuals had one or more identical genetic match, 16.2% of which had one or more same-collection genetic match and 4.9% had one or more genetic match among collections. AMOVA indicated that most genetic variation occurred within collections (90%; Φ_PT_ = 0.10; *P* ≤ 0.001; see Table 4). Results of Mantel tests (permutation *N* = 9999) indicated that a significant proportion of genetic variation was explained by collection site proximity (*R* = 0.09; *P* = 0.001) and elevation (*R* = 0.15; *P* = 0.001).

**Table 3:** Distance-Based Redundancy Analysis (DB-RDA) results by category.

Source	Trace	*F*	*P*
Local collections	14.3%	4.021	<0.002
Local collections (identical genetic matches removed)	12%	2.2	<0.002
Releases (MT-1, High Plains, Opportunity, Sherman)	31.3%	8.192	<0.002
‘Sandbergii’ releases (MT-1 and High Plains)	6.6%	2.204	<0.004
‘Sandbergii’ (Local Collections, MT-1, High Plains)	23.8%	5.31	<0.002
‘Sandbergii’ (pooled local collections, MT-1, High Plains)	3.8%	3.082	<0.002

Trace values indicate the percentage of variation explained for by source identities at the given *F* and *P* levels.

**Table 4:** Distribution of variation within and between *Poa secunda* source identities.

Source	Within % (est. var.)	Between % (est. var.)	Total est. var.	Φ_PT_	*P*
Local collections	90% (4.989)	10 (0.567)	5.556	0.102	≤ 0.001
Local collections (genetic matches removed)	93% (5.389)	7% (0.408)	5.796	0.070	≤ 0.001
Releases (MT-1, High Plains, Opportunity, Sherman)	57% (3.751)	43% (2.871)	6.622	0.434	≤ 0.001
‘Sandbergii’ releases (MT-1 and High Plains)	92% (5.575)	8% (0.487)	6.063	0.080	≤ 0.001
‘Sandbergii’ (local collections, MT-1, High Plains)	90% (5.116)	10% (0.587)	5.703	0.103	≤ 0.001
‘Sandbergii’ (pooled local collections, MT-1, High Plains)	93% (5.493)	7% (0.393)	5.886	0.067	≤ 0.001
Opportunity, MT-1, High Plains	70% (4.593)	30% (1.949)	6.543	0.298	≤ 0.001

Distribution of variance by *Poa secunda* source identities (collection or release) was determined by analysis of molecular variance (AMOVA).

### Variation in commercial *Poa secunda* releases

We analyzed releases (Opportunity, High Plains, MT-1, and Sherman) separately from local collections and found that 31.3% of variation was explained by release identity (DB-RDA; *F* = 8.192; *P* < 0.002). In analyses including only ‘Sandbergii’ variant releases (High Plains and MT-1), 6.6% of data variation could be explained by release identity (DB-RDA; *F* = 2.2; *P* < 0.004). No identical genetic matches were found within or between MT-1 and High Plains. Thirty-three percent of Opportunity individuals had one or more within-release genetic match. All Sherman individuals were genetically identical.

### Variation in commercial releases and local *Poa secunda*

**Fig 3:**
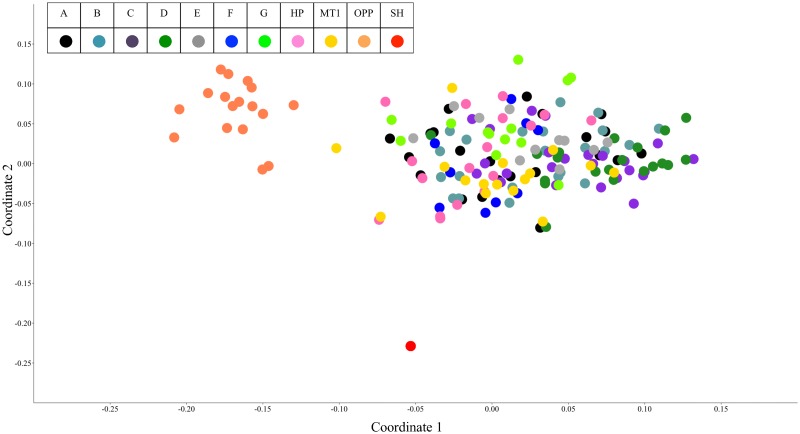
PCoA of local and commercial release *Poa secunda*. Principle coordinates analysis (PCoA) of all local collections and releases using a Jaccard similarity index, collection or release identity are indicated by dot color according to legend. The first axis explains 11.14% of the variation in the AFLP profiles and the second explains 8.08%, both axes describe 19.22% of variation.

We compared local collections, MT-1, and High Plains AFLP profiles and found that 23.8% of AFLP variance could be explained by collection site or release identity (*F* = 5.3; *P* < 0.002). However, when we pooled local collections, only 3.8% of the variance in AFLP profiles could be explained by release or local identity (*F* = 3.1; *P* < 0.002). Similar to local collections, this result suggested most of the variation occurs within rather than between ‘Sandbergii’ sources. Principle coordinates analysis explained 19.22% of the variation in local collections and releases in the first two axes, with 11.14% of variation explained by the first axis and 8.08% by the second axis (Fig 3). Analysis of molecular variance (AMOVA) of MT-1 and High Plains also showed that more genetic variance was within (92%) than between releases (8%) (Φ_PT_ = 0.080; *P* ≤ 0.001).

## Discussion

Genetic diversity of local *P*. *secunda* collections varied significantly among collection sites, and correlated with geographic distance and elevation. Variation among collection sites indicated spatial genetic structure at the scale of sampling. Our collections varied more strongly with elevation than space, suggesting that environmental conditions, such as temperature and precipitation, which co-vary with elevation at our study site, contribute to genetic structuring across the landscape. Most genetic variation was distributed within collections, consistent with the findings of Larson et al. [42]. These authors interpreted their results as being consistent with high levels of outcrossing. Although we cannot determine the frequency or isolate the specific influence of a given reproductive mode, our data suggest that there is localized genotypic variation.

We found identical genetic matches in our dataset, with most matches occurring within, rather than between, collections. Genetic matches varied between collections, ranging from 0 to 46% of individuals with one or more intra-collection genetic match. Although our analysis are insufficient to determine the relative frequency of reproductive mode, these results are not inconsistent with the variable frequency of apomixis in *P*. *secunda* demonstrated by Kellogg [10]. Inter-collection identical genetic matches occurred less often, with only 4.9% of individuals having one or more such match, a rate similar to our error threshhold. While intra-collection genetic matches occurred more frequently than inter-collection genetic matches, when they did occur it was usually between the most proximate collections (Fig 1). Our analyses, though limited in spatial scale, support a localized, mosaic patterning of genetic lines [17,57].

We evaluated population genetic structure to identify patterns relevant for the collection of representative local germplasm and the introduction of restoration plant materials. We found significant differences among collection sites. *Poa secunda* collections would best capture local diversity if sampled using guidelines typical for selfing or asexual species by emphasizing the collection of many individuals from large populations and many locations [27]. This mitigates the risk of over-sampling similar genotypes and also improves capture of site-specific genotypes.

Restoration plantings are often accomplished with commercial releases of native plants. We evaluated the High Plains, MT-1, Opportunity, and Sherman releases of *P*. *secunda* to compare them to one another and to wild populations represented by local collections. The four releases differed significantly from one another, consistent with phenotypic variation between *P*. *secunda* releases noted in previous studies [58,59]. High Plains and MT-1 diverged the least while Sherman and Opportunity differed from each other as well as High Plains and MT-1. Only Sherman and Opportunity had intra-release identical genetic matches, and the former constituted a single, shared genotype while 33% of Opportunity individuals were represented by one or more genetic matches, consistent with a high level of apomixis. We did not find genetic matches that included more than one release, as is expected given the distance among sources.

Variant identity likely drives the divergence of Opportunity and Sherman from the ‘Sandbergii’ variants [1,60]. We evaluated only a single release of each of the non-‘Sandbergii’ variants and they both have unique provenances, so we cannot quantify this divergence definitively. Sherman is apomictically fixed and represents a reproductively isolated subset of diversity within *P*. *secunda* [42]. Opportunity was sourced from remnant populations of ‘Nevadensis’ *P*. *secunda* on heavy metal contaminated, low pH sites and evaluated for performance at similarly degraded sites [46]. These sourcing and evaluation conditions constitute selective filters not shared by any other collection or release.

MT-1 was sourced more recently and in closer geographic proximity to local collections than High Plains, but neither was significantly more or less similar to local collections. High Plains was established from three source collections, but we did not find subgroupings as would be expected if the source populations were retained during selection and commercial increase. Opportunity was sourced from sites nearest to our local collections, but there was no evidence of genetic overlap, likely due to variant identity and/or sourcing and development history. We did not find any identical genetic matches between any of the releases and locally collected individuals.

Genetic variation in populations is desirable because it functions as a pool of adaptive capacity. Despite a strong theoretical basis [38,39], empirical evidence for genetic truncation resulting from plant materials development is limited. Ferdinandez et al. [61] found limited (8%) loss of genetic variation after two generations of seed increase in a multisite composite collection of the self-compatible *Elymus trachycaulus* ssp. *Subsecundus*. Fu et al. [62] examined the highly outcrossing *Bouteloua gracilis* (blue grama) and found no evidence for gene shifts after two generations of seed increase. We found comparable levels of variation in local (wild) collections and the ‘Sandbergii’ releases, High Plains and MT-1. The genetic diversity of the original High Plains and MT-1 source populations is unknown and the evaluation of genetic truncation is outside the scope of this study, but our analyses do not indicate that these releases lack variability compared to our local collections.

Facultative apomixis may confer genetic stability in diverse populations by slowing genetic equilibration to new environmental conditions [24], and may also mitigate the effects of agricultural selection [63]. Oversampling genotypes or collecting low-diversity populations would undermine this stability. Seeds of the low diversity Sherman and Canbar releases exemplify this risk, though no other *P*. *secunda* release or collection displays this level of genetic uniformity. Collections such as Sherman, that represent a fixed genotype, might displace other genotypes through “genetic swamping,” limiting overall population diversity in restoration plantings and impacting remnant, wild populations [30].

AFLPs generally represent neutral genetic variation, and therefore do not correspond to functional variation. Although genetic diversity is important for long-term adaptability, how genetic variation manifests as phenotype in restoration plantings determines plant establishment and persistence [33,41,64]. Concurrent with genetic differentiation among collections, previous studies found phenotypic differences as well. Herget et al. [58,59] found that survival, biomass, seed size, days to emergence, and competitiveness with *Bromus tectorum* differed between the High Plains release and our local *P*. *secunda* collections. Herget et al. [65] also identified differences in germination timing, early, and total root growth among our local collections, MT-1, High Plains and Opportunity. Along similar lines, Baughman et al. [66] compared local *P*. *secunda* collections with the Mountain Home release in the context of cheatgrass die-offs and found better performance in the local collections. Johnson et al. [63] found higher survival and greater leaf area in cultivars (of the variant ‘Sandbergii’) than in other collections in common gardens.

The closer we can get to evaluating restoration genetic concerns at their source, the better we can mitigate them. A clear understanding of how native plant species vary genetically in the landscape is essential. In the meantime, the use of commercial releases is common and widespread, so we must identify genotype and phenotype mismatches with natural populations, monitor their performance and persistence in restoration plantings, and incorporate them into the development of trait-based, plant material transfer zones.

## Supporting information

S1 Dataset *Poa secunda* binary AFLP profiles. (TXT)

S2 Dataset Site environmental data for locally collected *Poa secunda*. Latitude, longitude, insolation (watt hrs./sq. m), aspect and elevation (m) for all local *Poa secunda* collection sites.
(TXT)
